# Byproducts (Flour, Meals, and Groats) from the Vegetable Oil Industry as a Potential Source of Antioxidants

**DOI:** 10.3390/foods11030253

**Published:** 2022-01-18

**Authors:** Mihaela Multescu, Ioana Cristina Marinas, Iulia Elena Susman, Nastasia Belc

**Affiliations:** 1 National Institute of Research and Development for Food Bioresources—IBA Bucharest, 020323 Bucharest, Romania; iulia.susman@bioresurse.ro (I.E.S.); nastasia.belc@bioresurse.ro (N.B.); 2 Faculty of Biotechnology, University of Agronomic Sciences and Veterinary Medicine of Bucharest, 011464 Bucharest, Romania; 3 Research Institute of the University of Bucharest—ICUB, 050095 Bucharest, Romania; ioana.cristina.marinas@gmail.com

**Keywords:** byproducts, vegetable oil industry, phenolics, flavonoids, antioxidant activity, photochemiluminescence

## Abstract

The present study presents the use of photochemiluminescence assay (PCL) and 2,2 diphenyl-1-picryl-hydrazyl (DPPH), 2,2′-azinobis-(3-ethylbenzothiazoline-6-sulfonic acid (ABTS), the ferric reducing antioxidant power (FRAP), and cupric ion reducing antioxidant capacity (CUPRAC) methods for the measurement of lipid-soluble antioxidant capacity (ACL) of 14 different byproducts obtained from the vegetable oil industry (flour, meals, and groats). The research showed that the analyzed samples contain significant amounts of phenolic compounds between 1.54 and 74.85 mg gallic acid per gram of byproduct. Grape seed flour extract had the highest content of total phenolic compounds, 74.85 mg GAE/g, while the lowest level was obtained for the sunflower groats, 1.54 mg GAE/g. DPPH values varied between 7.58 and 7182.53 mg Trolox/g of byproduct, and the highest antioxidant capacity corresponded to the grape seed flour (7182.53 mg Trolox/g), followed by walnut flour (1257.49 mg Trolox/g) and rapeseed meals (647.29 mg Trolox/g). Values of ABTS assay of analyzed samples were between 0 and 3500.52 mg Trolox/g of byproduct. Grape seed flour had the highest value of ABTS (3500.52 mg Trolox/g), followed by walnut flower (1423.98) and sea buckthorn flour (419.46). The highest values for FRAP method were represented by grape seed flour (4716.75 mg Trolox/g), followed by sunflower meals (1350.86 mg Trolox/g) and rapeseed flour (1034.92 mg Trolox/g). For CUPRAC assay, grape seed flour (5936.76 mg Trolox/g) and walnut flour (1202.75 mg Trolox/g) showed the highest antioxidant activity. To assess which method of determining antioxidant activity is most appropriate for the byproducts analyzed, relative antioxidant capacity index (RACI) was calculated. Depending on the RACI value of the analyzed byproducts, the rank of antioxidant capacity ranged from −209.46 (walnut flour) to 184.20 (grape seed flour). The most sensitive methods in developing RACI were FRAP (r = 0.5795) and DPPH (r = 0.5766), followed by CUPRAC (r = 0.5578) and ABTS (r = 0.4449), respectively. Strong positive correlations between the antioxidant capacity of lipid-soluble compounds measured by PCL and other methods used for determining antioxidant activity were found (r > 0.9). Analyses have shown that the different types of byproducts obtained from the vegetable oil industry have a high antioxidant activity rich in phenolic compounds, and thus their use in bakery products can improve their nutritional quality.

## 1. Introduction

During the process of obtaining vegetable oils, considerable amounts of waste and byproducts are generated. These byproducts from the vegetable oil industry are important due to their high value-added substances, and they represent an excellent source of bioactive components, such as antioxidants. Byproducts such as flour, meals, and groats resulting from the vegetable oil industry are considered economic resources due to the antioxidant compounds, which have attracted interest in making functional products with a higher nutritional value, satisfying consumer demand for such products [1].

Sea buckthorn berries are rich in substances with biological activity [2]. Sea buckthorn is rich in nutrients and antioxidants, so it is used as a dietary supplement [3]. Ghendov-Mosanu et al. [2] have determined the characteristics of sea buckthorn flour and investigated its effects on sensory, physicochemical, and antioxidant properties. The results obtained showed that sea buckthorn flour is a good source of ascorbic acid, polyphenols, and flavonoids. This byproduct added in different concentrations in wheat bread has extended the shelf life to 72 h and has improved antioxidant activity. Increasing the concentration of sea buckthorn flour in wheat bread was directly proportional to its benefits. Hemp is rich in protein, fat, carbohydrates, and fiber. It contains significant amounts of macroelements, such as P, K, Mg, Na, and Ca. Hemp flour is a good source of bioactive compounds, especially polyphenols [4]. Rusu et al. [4] have nutritionally characterized the bread with the addition of hemp flour in different concentrations. Studies have shown that the addition of hemp flour to bread improves the nutritional properties.

Walnuts are a significant source of vegetable protein and amino acids. They contain over 50% oil rich in polyunsaturated fatty acids. Walnuts are an excellent source of phytochemicals, such as phenolic compounds, carotenoids, and tocopherols [5]. Almoraie et al. [6] determined the possibility of using walnut flour in the production of wheat bread. Bread samples with different concentrations of walnut flour have been shown to have a higher nutritional value than bread samples made from 100% wheat flour. Grape seed flour contains a significant number of polyphenols and presents high antioxidant capacity [7]. This byproduct can be an alternative in the production of various foods due to a high content of dietary fiber. Previous studies have shown that grape seed flour has been used in many products, such as cookies, pancakes, butter biscuits, and bread [8].

Sunflower has many nutritional components. Examples are sunflower flour, meal, groats, etc. [9]. Sunflower meal can be used in a wide range of bakery products due to the high concentration of antioxidants. Byproducts such as sunflower meal represent an excellent source of protein, essential amino acids, and fiber. It contains essential amino acids, vitamin B, and minerals, and has a high antioxidant property [10]. Grasso et al. [11] observed that sunflower meal could be used for the nutritional improvement of muffins. Rapeseed’s meal is rich in phenolic compounds, tocopherols, vitamins B, calcium, magnesium, and presents high antioxidant activity [12]. Along with rapeseed meal, black sesame meal is a potential source of polyphenolics with high antioxidant status [13]. Flax seeds contain a high level of oil and are rich in polyunsaturated fatty acids. They are also an important source of soluble and insoluble fiber [14]. Numerous phytochemical compounds with antioxidant activity are found in flax seeds, including phenolic acids, flavonoids, and lignins [15]. Pourabedin et al. [16] observed that adding flaxseed flour to toast bread increases phenolic compounds. Milk thistle is known for its high content of bioactive compounds, mainly flavonoids with a powerful antioxidant character [17]. Milk thistle seeds contain the largest number of active substances, about 70–80% of silymarin flavonolignans and about 20–30% of substances with polyphenolic structures [18].

The byproducts (meal) obtained in the vegetable oils industry contain phenolic compounds with different chemical structures, such as tocopherols, carotenoids, flavonoids, lignins, phenolic acids, and tannins with high values of antioxidant capacity [19]. These are natural and cheap sources of antioxidants that could replace synthetic additives (BHT or BHA) [19]. It is known that antioxidants play an important role in preventing many diseases, such as cancer and cardiovascular disease [20]. The main biologically active compounds of the byproducts obtained from the vegetable oil industry are polyphenols and flavonoids. Phenolic compounds are a broad group of secondary metabolites that are spread throughout the plant. Polyphenolics have special properties for human health, including anti-inflammatory activity, enzyme inhibition, antimicrobial, antiallergic, reducing the risk of cardiovascular disease, and cytotoxic antitumor activity [21]. Antioxidants such as polyphenols are considered possible protective agents, reducing the oxidative damage caused by reactive oxygen species in the human body and delaying the progression of many chronic diseases, as well as the oxidation of low-density lipoproteins (LDL), which play an important role in atherosclerosis [22]. One of the major causes of atherosclerosis is the high cholesterol. Flavonoids represent a class of phenolic compounds, which are found naturally in plants. These bioactive compounds are also found in a variety of nutraceutical, pharmaceutical, medicinal, and cosmetic applications. This is attributed to their antioxidant, anti-inflammatory, antimutagenic, and anticarcinogenic properties, along with their ability to modulate the key function of cellular enzymes. Research on flavonoids has received an additional boost with the discovery of a low rate of cardiovascular mortality and the prevention of cardiovascular disease [23].

The aim of this study was to determine the total polyphenolic and flavonoid content, as well as to evaluate the antioxidant activity by different methods (DPPH, FRAP, CUPRAC, ABTS) and the antioxidant capacity by PCL-ACL of various byproducts obtained from the vegetable oil industry. It is necessary to find alternative strategies for the use of these byproducts in order to avoid their impact on the environment and to increase the profitability of plant resources. The main attraction of these byproducts obtained in the vegetable oil industry is the possibility to use them in the production of food products with high nutritional value.

## 2. Materials and Methods

### 2.1. Reagents and Standards

2,2-Diphenyl-1-picrylhydrazyl (DPPH), 2,2′-azino-bis(3-ethylbenzothiazoline-6-sulphonic acid) diammonium salt (ABTS), ferric reducing antioxidant potential (FRAP), 2,4,6-tripyridyl-s-triazine (TPTZ), (+)-catechin, gallic acid, and Trolox (6-hydroxy-2,5,7,8-tetramethylchroman-2-carboxylic acid) were purchased from Sigma Chemical Co. (Switzerland). Folin–Ciocalteu’s phenol reagent was purchased from Merck (Germany). All chemicals used were of analytical grade. Standard solutions were prepared with distilled deionized water.

### 2.2. Byproduct Materials

The following types of byproducts were considered: sea buckthorn flour, hemp flour, walnut flour, grape seed flour, rapeseed meals, sunflower meals, black sesame meals, red grape seed meals, golden flax meals, thistle meals, sesame groats, thistle groats, coriander groats, sunflower groats. The byproducts were obtained from the vegetable oil industry and provided by the Association of Operators in Organic Farming Bio-Romania (Romania). The samples were ground using a laboratory mill and kept in closed jars in a refrigerator at 4 °C.

### 2.3. Qualitative Analysis by ATR-FTIR

The FTIR spectrum for byproducts and dry extracts was recorded at room temperature using the Cary 630 FTIR Spectrometer in ATR mode (Agilent Technologies Inc., Santa Clara, CA, USA). The chosen measurement range was 4000–650 cm^−1^, number of scans 400, resolution 4 cm^−1^. The FTIR spectra of dry extract were produced on the alcoholic extract of byproducts dried previously in an oven at 25 °C (for 24 h) [24].

### 2.4. Extraction Procedure

An amount of 0.2 g of byproduct was weighed and brought into 20 mL of ethanol 96 %. The extracts were obtained by the ultrasound-assisted extraction method involving frequencies ranging from 20 kHz to 2000 kHz for 1h at room temperature. Then, the extracts were centrifuged for 10 min at 10,000 rpm to remove the secondary materials [25].

### 2.5. Determination of Phenol Content

Total phenol content was determined by the Folin–Ciocalteu method [26]. A total of 50 μL of extract was mixed with 10 µL Folin–Ciocalteu reagent, 90 µL distilled water, and 10 µL of saturated sodium carbonate. The 96-well plates were allowed to stand in the dark for 60 min for color development. Absorbance was measured at 765 nm using a FlexStation 3 UV-Vis (Molecular Devices, GA, USA) Spectrophotometer. A standard curve was prepared by using different concentrations (10–50 μg/mL) of gallic acid in the same condition with samples (R^2^ = 0.9966). Total phenolic content was expressed as mg gallic acid equivalent/g of byproduct (mg GAE/g).

### 2.6. Determination of Flavonoid Content

Total flavonoid content (TFC) was assessed through the AlCl_3_ method described by Woisky and Salatino [27]. Briefly, 0.1 mL sample/standard solution was mixed with 0.1 mL 10% sodium acetate and 0.12 mL 2.5% AlCI_3_, the final volume being adjusted to 1 mL with 70% ethanol. The samples were then vortexed and incubated in the dark for 45 min. The absorbances were measured at 430 nm. A standard curve was plotted by using different concentrations (5–200 μg/mL) of quercetin (R^2^ = 0.9980). Total flavonoid content was expressed as mg quercetin equivalent/g of byproduct (mg QE/g).

### 2.7. Determination of Antioxidant Activity through DPPH, CUPRAC, FRAP, and ABTS Methods

DPPH radical scavenging activity was determined based on the reduction in DPPH radical, according to Culetu et al. [28], with slight modifications. The reaction mixture consisted of 1 mL of sample and 6 mL of DPPH radical solution, which was incubated for 20 min in the dark. Then, the absorbance was measured at 517 nm. Antioxidant activity was calculated using a calibration curve (0.0156–0.0625 μg/mL) obtained with Trolox (R^2^ = 0.9998). The results were expressed in mg Trolox/g of byproduct.

The CUPRAC method is based on the reduction of a cupric complex, neocuproin, by antioxidants in copper form. Copper ion reduction was performed according to a method described by Celik et al. [29]: 60 μL of sample/standard solutions of different concentrations were mixed with 50 μL CuCl_2_ (10 mM), 50 μL neocuproin (7.5 mM), and 50 μL ammonium acetate buffer 1 M, pH = 7.00. After 30 min, the absorbance was measured at 450 nm. The stock Trolox solutions required for the calibration curve were 2 mM, and the working concentrations were between 0.125 and 2.0 mM (R^2^ = 0.9977). The results were expressed in mg Trolox/g of byproduct.

FRAP assay—the determination of the antioxidant capacity of iron reduction was performed by the method described by Thaipong et al. [30]. The stock solutions included 300 mM acetate buffer (3.1 g C_2_H_3_NaO_2_ 3H_2_O and 16 mL C_2_H_4_O_2_), pH 3.6, 10 mM 2,4,6-tripyridyl-s-triazine (TPTZ) solution in 40 mM HCl, and 20 mM FeCl3 6H2O solution. The fresh working solution was prepared by mixing 25 mL acetate buffer, 2.5 mL TPTZ solution, and 2.5 mL FeCl_3_ 6H_2_O solution, and then warming at 37 °C before using. After incubation, the absorbance was read at 593 nm. A 1 mM Trolox stock solution was used to plot the calibration curve, the concentration ranging between 25 and 250 µM Trolox (R^2^ = 0.9962). The results were expressed in mg Trolox/g of byproduct.

Trolox equivalent antioxidant capacity (TEAC) assay was performed according to Re et al. [31] with slight modifications. A stable stock solution of ABTS^+^ was produced by mixing a solution of 7 mM ABTS in 2.45 mM potassium persulphate. Then, the mixture was left standing in the dark at room temperature for 12–16 h before use. An ABTS^+^ working solution was obtained by dilution with ethanol to an absorbance of around of 0.70. The reaction mixture consisted in 20 μL of sample/standard and 180 μL of ABTS^+^ working solution and was incubated 30 min in the dark. The standard curve was linear between 20 and 200 µM Trolox (R^2^ = 0.9975). The results were expressed in mg Trolox/g of byproduct.

### 2.8. Photochemiluminescence Assay

An amount of 0.05 g of samples was mixed with 30 mL methanol for 3 h [32]. The extractions were performed in tightly closed plastic tubes, vortexed, and centrifuged at 20 °C. The extraction was made in triplicate. The scavenging activity of byproduct samples was evaluated by a photochemiluminescence (PCL-ACL) method in which superoxide radical anions (O_2_−) are generated from luminol. The extracts were dissolved in methanol. The reactions were carried out using kits for the determination of antioxidant capacity of lipid-soluble substances (Analytik Jena, Jena, Germany), mixing 2300 μL of methanol (reagent 1), 200 µL of buffer solution (reagent 2), 25 µL of luminol (reagent 3), and 10 µL of sample. Measurement was performed on a Photochem device with PCLsoft software (Analytik Jena). Trolox was used to prepare the calibration curve. The results are expressed as mg of Trolox equivalents per g of extract.

### 2.9. Statistical Analysis

Antioxidant assays were performed in at least three repetitions. Results are presented as means ± standard deviation (SD). To determine the relation between results of total phenolics and antioxidant assays, the Pearson correlation was used.

## 3. Results and Discussion

### 3.1. Qualitative Analysis by ATR-FTIR

**Figure 1:**
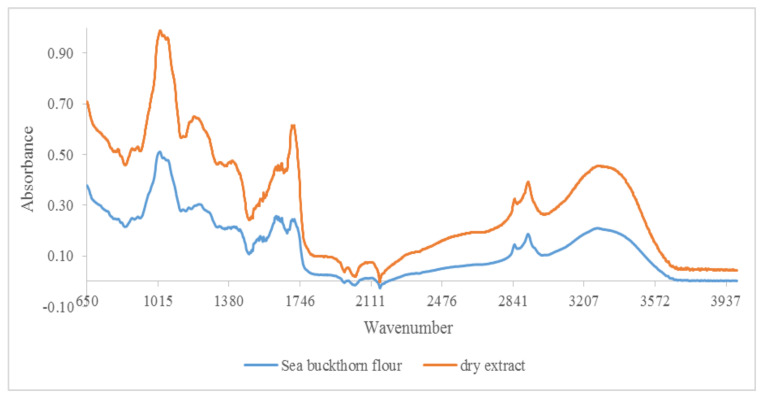
FTIR spectra for sea buckthorn flour and its dry extract in the region between 650 and 4000 cm^−1^.

Qualitative analysis of FTIR spectra provides a characteristic signature present for specific bonds of interest in antioxidants (phenolic, phenolic acids, flavonoids, hydroxycinnamic acids, etc.) by the presence of their molecular vibrations (stretching, bending, and torsion of chemical bonds) [33,34]. Therefore, FTIR spectra represent a molecular footprint of samples for both byproducts and dry extracts obtained by evaporation at room temperature in the dark. Because the amount of some of the dry extracts were low, it was not possible to perform FTIR spectra for all extracts. Figure 1 shows the comparative spectra for both byproducts and dry extracts to highlight specific bands of phenolic compounds.

**Figure 2:**
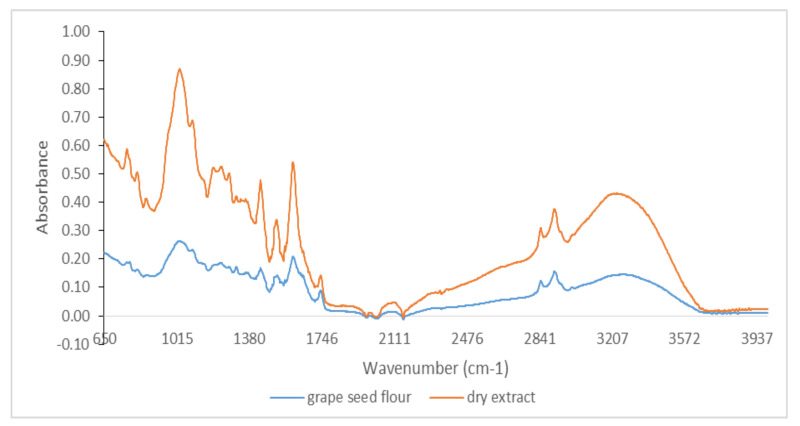
FTIR spectra for grape seed flour and its dry extract in the region between 650 and 4000 cm^−1^.

**Figure 3:**
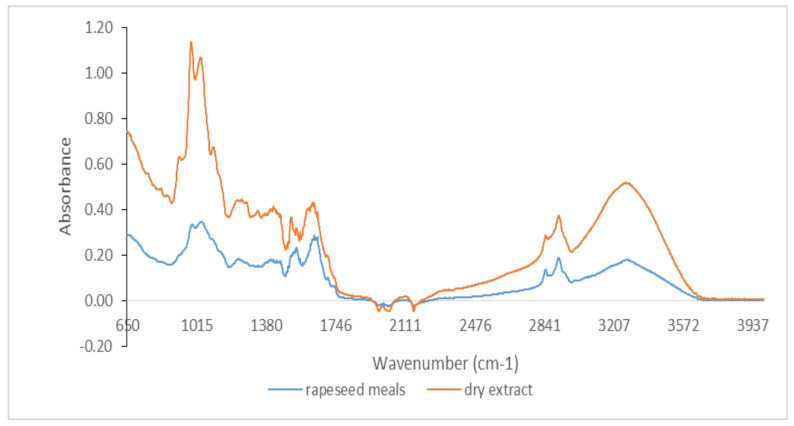
FTIR spectra for rapeseed meals and its dry extract in the region between 650 and 4000 cm^−1^.

**Figure 4:**
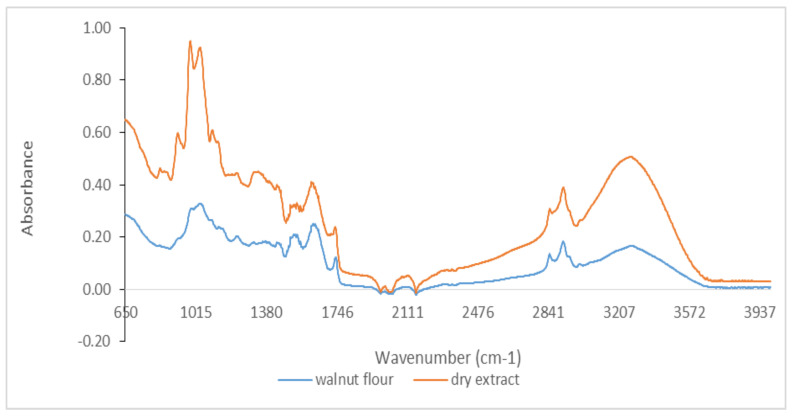
FTIR spectra for walnut flour and its dry extract in the region between 650 and 4000 cm^−1^.

It was possible to distinguish several peaks, which correspond to the functional groups and vibration modes of the polyphenolic components. The wide band between 3220 and 3249 cm^−1^ corresponded to the OH stretching modes and it could be attributed to polysaccharides and/or lignins. The peak at 3010 cm^−1^ was related to the C–H stretching vibration of the cis-double bond (=CH) groups. The asymmetric and symmetrical stretching vibrations of the CH_2_ groups were found at 2924 cm^−1^ and 2853 cm^−1^, respectively, in both dry extract and byproduct for hemp flour, seed flour harrows, sunflower meal, and sesame meal. They were mainly associated with the hydrocarbon chains of lipids or lignins [35]. The spectral band at 1744 cm^−1^ (for hemp flour, sunflower meal, and sesame meal) and the shoulder band at 1719 cm^−1^ (for sea buckthorn flour and walnut flour) was attributed to the absorption of C=O bonds of the ester groups and was related to the presence of fatty acids and glycerides, as well as pectins and lignins [36]. The bands around 1600 cm^−1^ (hemp flour, walnut flour, grape seed flour, and rapeseed meals) were associated with the extent of the carboxyl group and aromatic ring, for example, in pectins and phenolic compounds [37], but also with the bending vibrations of the OH groups. The footprint region from 1500 to 800 cm^−1^ was very rich in peaks from different ways of stretching, bending, swinging, scissoring, and twisting. This region was difficult to analyze due to its complexity, providing important information about organic compounds, such as carbohydrates, alcohols, and organic action, present in the samples. Aromatic C–C extending to ~1520 (seed flour harrows and rapeseed meals) and ~1443 cm^−1^ (walnut flour, grape seed flour, and sesame meal) was related to phenolic compounds [38]. Methyl radical bent in the plane at 1377 cm^−1^ (hemp flour, sunflower meal, and sesame meal) and C–O extending at ~1035 cm^−1^ (walnut flour, grape seed flour, and rapeseed meals) were related to polysaccharide structures [34,36,37]. The peak at 1143 cm^−1^ (identified for hemp flour and sesame meals) corresponded to the aromatic extent of C–H. The band at 765 cm^−1^ has been assigned to benzene ring 1,3-disubstituted, specific for phenolic compounds [38]. Therefore, in the studied extracts, specific bands of phenolic compounds were identified (Figure 1, Figure 2, Figure 3 and Figure 4).

### 3.2. Amount of Phenolic Compounds and Flavonoid Compounds

The amount of total phenolic and flavonoid content in the byproduct extracts is shown in Table 1.

The different byproducts from the vegetable oil industry contain significant amounts of phenolic compounds. Analyzed extracts contained between 1.54 and 74.85 mg GAE per gram of byproduct. Grape seed flour extract resulted in the highest amount of total phenolic compounds, 74.85 mg GAE/g. The lowest level was obtained for the sunflower groats, 1.54 mg GAE/g. These amounts were comparable with results described in the literature for other extracts of plant products. The byproducts of the wine industry, mainly fresh and fermented grape pomace, represent a potential source of natural phenolic substances. Grape seeds are richer in polyphenolic compounds and have a higher antioxidant activity than grape skins. The content of extractable phenolic compounds from seeds represents almost 70% of the total extractable phenols from grapes [39]. Rajakumari et al. [40] formulated a nanodispersion containing grape seed extract and analyzed its release profile and the antioxidant potential of the prepared formulations. They concluded that the formulations present a high antioxidant scavenging due to the content of phenolic compounds. The resulting value of total polyphenol content of the tested grape seed flour is higher than those obtained by Antonic et al. [8], 5.58 mg GAE/g. Lutterodt et al. [41] analyzed flours made from cold-pressed seeds of two grape cultivars. The obtained values were 5.93 and 6.66 mg GAE/g. In the same study, the results of TPC for another two cultivars were 67.9 and 89.6 mg GAE/g. These indicate that the TPC content is highly dependent on the cultivar.

Gallic acid, 3,4-dihydroxybenzoic acid, (+)-catechin, 1,2-dihydroxybenzene, and syringic acid were the major phenolic compounds in hemp seed [42]. Phenolic content of hemp flour was 10 times higher than those obtained by Ertas et al. [43], who reported that raw hemp flour contains 0.405 mg GAE/g. In contradiction with our results, Mikulec et al. [44] reported that hemp flour is characterized by a polyphenol content of 0.98 mg GAE/g. Walnuts are recognized for their high antioxidant capacity, being associated with a high phenolic content. Phenolic compounds, hydrolyzable tannins, and flavanols are the major components in walnut flour [45]. Similar to our results, Burbano and Correa [46] reported that the total phenolic content of walnut flour is 10.9 ± 0.3 mg GAE/g. Santos et al. [47] studied the effect of roasting conditions on the composition and antioxidant properties of defatted walnut flour and they observed that walnut flour contains 20 mg GAE/g. Labuckas et al. [48] analyzed three walnut flour varieties with respect to total phenolic content (TPC). They observed that TPC varied between 16.3 and 23.7 mg GAE/g, the results being higher than those obtained in this study.

Rapeseed meal is the byproduct of the rapeseed de-oiling process. Rapeseed contains the greatest number of phenolic compounds compared with other oilseed plants. The most significant phenolic compounds in rapeseed are sinapic acid derivatives, such as sinapine [49]. The rapeseed meal phenolic isolates contained between 0.42 and 6.94 mg GAE/g depending on the extraction solvent used [49]. According to Yang et al. [50], total phenolic compounds in rapeseed meals ranged from 38.50 to 63.95 mg GAE/g dry weight. These values are higher than those reported in the present study (11.24 mg GAE/g).

It is known that sunflower seeds are rich in phenolic compounds and the total phenolic content was in the range of 10–42 mg/g according to Kreps et al. [19]. Zilic et al. [51] investigated the total polyphenol content in three sunflower genotypes. They found that the polyphenol content ranged between 14.68 and 18.24 mg GAE/g for sunflower seed and 16.28 and 20.13 mg GAE/g for sunflower kernel, respectively. Chlorogenic and caffeic acids represented 70% of phenolic compounds in sunflower flour [19].

Elleuch et al. [52] have performed a detailed study on the chemical composition and the importance of each component in the seed coat. They studied the total phenolic content of raw sesame seed (0.88 mg GAE/g) and the raw seed coat (0.60 mg GAE/g). Sesame seed coat showed a relatively high polyphenol content (9.9 mg/g of seed coat dry matter) [53]. Compared with black sesame meals (3.88 mg GAE/g), it was observed that seed coat contains a higher amount of total polyphenols. Barthet et al. [54] determined antioxidant activity of flaxseed meal components. They reported that the amount of total phenolics extracted by the various solvents decreased from methanol to water extracts, the results varying between 98.2 mg GAE/g and 68.2 mg GAE/g, respectively. Our results are lower than those obtained by Barthet et al. [54].

The total phenolic content of milk thistle genotypes ranged from 2.06 to 3.60 mg GAE per g [17]. Stancheva et al. [55] reported that the total phenolic content of thistle was between 2.58 and 3.91 mg GAE/100 g. Phenolic content was higher in thistle meals (7.97 mg GAE/g) and thistle groats (8.39 mg GAE/g) than for milk thistle.

The flavonoid content represents approximately 80.56% of the total polyphenols for sea buckthorn flour extract, 88.03% for hemp flour extract, 55.05% for walnut flour extract, 68.46% for grape seed flour extract, 84.16% for rapeseed meals, 67.69% for sunflower meal extract, 32.41% for sesame groats, and 59.48% for thistle groats (Table 1). The results were correlated with the literature data; for example, the percentage of flavonoids was similar to that obtained by Nilova and Malyutenkova [56] for sea buckthorn flour extract (89.5%), and, for the hemp flour extract, the flavonoids content was significantly higher than those present in the literature, 0.29 mg catechin equivalent/g [42,57]. For walnut flour, a higher concentration of flavonoids was obtained than in Santos et al. [47]. Depending on the extraction solvent used, the content of flavonoids in rapeseed and sunflower varied between 0.74 and 4.19 mg catechin/g and 1.45 and 12.03 mg catechin/g, respectively [20]. In comparison with our results, rapeseed meal (11.24 mg QE/g) presented higher flavonoid content. Sunflower meals (11.70 mg QE/g) contain lower flavonoid levels than metanolic extract analyzed by Matthaus [20].

The variation in TPC and TFC could be due to the varietal differences, climate, harvest time, and other factors that affect the nutritional quality of the plants [58]. Moreover, the content of TPC and TFC depends on the extraction solvent. Khalil et al. [59] concluded that the extraction solvent of total phenolic contents and total flavonoid contents of pomegranate peel extracts is methanol, followed by ethanol and ethyl acetate. The vegetable oil industry generates substantial amounts of phenolic-rich byproducts, which could be valuable natural sources of antioxidants.

### 3.3. Comparison of the Antioxidant Activity of Selected byproducts

The response of antioxidants to different radical or oxidant sources may be different. Thus, no single method accurately reflects the mechanism of action of all radical sources or all antioxidant compounds in a complex system [60].

Evaluation of antioxidants in food is of great importance in today’s context. Estimation of total antioxidant using different assays is important to get the overall antioxidant potential of any food matrix. The total antioxidant activity of the 14 byproducts obtained from the vegetable oil industry was estimated using four in vitro assays, namely, DPPH, ABTS, FRAP, and CUPRAC. The antioxidant capacity through PCL was also determined. The measurements of DPPH, ABTS, FRAP, and CUPRAC values are presented in Table 2

These analyses can allow the identification and selection of byproducts in the vegetable oil industry that favor the enrichment of bakery products in antioxidants, which could lead to an increase in the quality of these products.

The total antioxidant activity using the DPPH method of tested samples was between 7.58 and 7182.53 mg Trolox/g of extract. Grape seed flour presented the highest antioxidant activity (7182.53 mg Trolox/g), while the value of DPPH in sesame groats was the lowest (7.58 mg Trolox/g). Scavenging activity of extracts of grape seed flour and walnut flour (1257.49 mg Trolox/g) were significantly higher than that of sesame groats; thus, phenolic compounds of grape seed flour and walnut flour exhibited the strongest DPPH scavenging potency. Grape seeds are rich in gallic acid, vanilla acid, caffeic acid, ferulic acid, p-coumaric acid, chlorogenic acid, rutin, and quercetin [61]. According to Ross et al. [62], the DPPH radical scavenging ability of defatted grape seed flour was 0.67 mg Trolox/g dry matter, lower than the result from this study. Ross et al. [62] determined the effect of heating time and temperature on the DPPH antioxidant activity of 70% ethanol extracts of grape seed flour and they observed that, at a temperature of up to 150 °C, the values of DPPH activity of the grape seed flour did not significantly change. At a temperature above 180 °C, significant decreases in antioxidant activity were observed.

Walnut kernels are rich in gallic acid, caffeic acid, chlorogenic acid, ferulic acid, synaptic acid, salicylic acid, and ellagic acid [63]. Walnuts are recognized for their high antioxidant capacity when compared with other nuts, being normally associated with this property with a high phenolic content [64]. Studies have shown that caffeic acid and gallic acid exhibited high DPPH scavenging activities, 89.4% and 88.5%, respectively [65]. Sroka and Cisowski [66] confirmed the strong activity of gallic acid and pyrogallol on DPPH scavenging tests, 75% and 79.5%, respectively. Trandafir et al. [62] analyzed the effect of different solvents and extraction methods on antioxidant activity of full-fat and defatted walnut kernel. The results showed that values of antioxidant activity ranged between 17.25 and 49.25 mg Trolox/100g. They concluded that antioxidant capacity of full-fat walnut kernel and defatted walnut kernel is influenced by extraction method.

Chlorogenic and caffeic acids represent almost 70% of phenolic compounds in sunflower flour [67]. Numerous studies have demonstrated that sunflower meal presents high antioxidant capacity, which could be beneficial for further technological utilization [68]. Grasso et al. [69] investigated defatted sunflower seed flour and they reported an antioxidant radical scavenging value of 4.5 mg Trolox/g, lower than our concentration reported in the preset study. The antioxidant activity of phenolic acids and their esters depend partly on the number of hydroxyl groups in the molecule [70], which explains the high DPPH scavenging activity of caffeic acid and gallic acid with three hydroxyl groups. Kikuzaki et al. [71] also have reported similar compounds of scavenging ability towards DPPH radicals: caffeic acid, sinapic acid, ferulic acid, and p-coumaric acid. Chlorogenic and isochlorogenic acids, which are derivatives of caffeic and quinic acid, exhibited strong antioxidative properties, 93% and 86%, respectively [66]. Ferulic acid showed a DPPH scavenging capacity of 39.5%, having in its structure a single methoxy group in the meta position [65].

The values of DPPH reported in this study were higher for meal or flour than for groats. This is because, in all meal or flour samples, the polyphenol content was higher than in the groats, except black sesame meal and thistle meal, where the level of phenolic compounds was higher in the groats. It is generally predicted that DPPH radical scavenging activity is strongly affected by the content of phenolic compounds [72].

Because the contribution of the phenolic compounds to the overall antioxidant capacity is different, a correlation analysis was performed (Table 3). The results showed a strong positive correlation of TPC with antioxidant activity on DPPH radical, with a correlation coefficient of 0.9927. Moreover, a high correlation was recorded between DPPH and TFC (0.9811).

The antioxidant capacity is measured as the ability of compounds to decrease the color reacting with ABTS^+^ radical and expressed relative to Trolox [73]. It is known that antioxidant activity depends on the content of the phenolics and the number and position of the hydroxyl groups of the aromatic ring binding site and the type of substituent. Similar to DPPH assay, the strongest antioxidant activity in the ABTS method was obtained by phenol with three hydroxyl groups, such as gallic acid and pyrogallol [74].

Values of ABTS^+^ of analyzed byproducts ranged from 0 to 3500.52 mg Trolox/g. Grape seed flour had the highest value of ABTS assay (3500.52 mg Trolox/g), followed by walnut flower (1423.98) and sea buckthorn flour (419.46). Ross et al. [62] analyzed a sample of defatted grape seed flour and they observed a TEAC value of 4.68 mg Trolox/g dry matter, lower than our result. Grape seed contains a high amount of polyphenols with three hydroxyl groups, mainly flavonoids, including gallic acid, the monomeric flavan-3-ols catechin, epicatechin, gallocatechin, epigallocatechin, and epicatechin 3-O-gallate, and procyanidin dimers, trimers, and more highly polymerized procyanidins [75]. Moreover, the walnut flour contains a great content of phenolic compounds, such as hydrolyzable tannins. They are responsible for a high antioxidant activity in walnut flour. According to Pellegrini et al. [76], antioxidant capacity of walnut extracts was 119.91 μmol Trolox/g dry matter by ABTS method. Antioxidant capacity of extracts of the whole walnut determined by Arranz et al. [77] was 165.18 μmol Trolox/g dry matter. After the sample was defatted with petroleum ether, the antioxidant capacity was higher than whole walnut at 211.85 μmol Trolox/g dry matter. The authors concluded that the high fat content of walnut interferes in the determination of antioxidant capacity [77]. Seventeen phenolic acids were identified in sea buckthorn berries. Salicylic acid was the predominant phenolic acid in berries. Small quantities of p-hydroxybenzoic acid were detected [78]. Cyclic spermidine-alkaloid, feruloyl choline, kaempferol, and sinapine were identified as the main phenolic compounds in rapeseed meals [50].

The ABTS values for golden flax meals (12.13 mg Trolox/g), sesame groats (15.77 mg Trolox/g), and coriander groats (9.37 mg Trolox/g) were the lowest, suggesting small quantities of phenolic compounds. Barthet et al. [54] analyzed two samples of golden flax meals and brown flax meals. They reported ORAC values of 2.44 mg Trolox/g and 2.33 mg Trolox/g, respectively.

The correlation between TPC and TFC with ABTS method is shown in Table 3. The correlation coefficients in these cases are 0.9660 and 0.9477, respectively.

A wide range in FRAP values was observed among the analyzed byproducts, showing values of 34.46–4716.75 mg Trolox/g of byproduct. The highest value of FRAP was presented by grape seed flour (4716.75 mg Trolox/g), followed by sunflower meals (1350.86 mg Trolox/g) and rapeseed meals (1034.92 mg Trolox/g). Gougoulias and Mashev [79] analyzed 14 grape seeds varieties. The results of FRAP activity were lower than those presented in this article and ranged between 0.15 and 0.20 mg Trolox/g dry weight. In contradiction with our results, Antonic et al. [8] analyzed grape seed flour with respect to antioxidant activity and observed that FRAP activity was 0.056 mg Trolox/g. Ross et al. [62] found that the FRAP value of defatted grape seed flour was 0.93 mg Trolox/g dry matter. Szydłowska-Czerniak et al. [12] evaluated antioxidant capacity of rapeseed meal. Results ranged between 0.003 and 0.025 mg Trolox/g. Gvozdenac et al. [80] tested seed, hull, and kernel of different sunflower hybrids. The results of antioxidant activity (FRAP) in seed ranges between 76.5 and 83.0 mg Trolox/g, in hull between 4.5 and 8.4 mg Trolox/g, and in kernel between 116.1 and 1117.3 mg Trolox/g. According to Arranz et al. [77], antioxidant activity of aqueous organic extracts of defatted walnut through FRAP method was 114.92 μmol Trolox/g dry matter.

CUPRAC antioxidant assay delivered values in the range of 62.45–5936.75 mg Trolox/g. Grape seed flour presented the highest CUPRAC value (5936.76 mg Trolox/g), while the lowest value was obtained by sesame groats (62.45 mg Trolox/g). According to Bogoeva et al. [81], the antioxidant activity of 70% ethanolic extract of grape seed flour was evaluated and they obtained a CUPRAC value of 344.78 mg Trolox/g, lower than our result. Grasso et al. [69] investigated defatted sunflower seed flour with respect to antioxidant activity. They observed that defatted sunflower seed flour had higher antioxidant capacity measured by DPPH and CUPRAC assays compared to wheat flour. The CUPRAC value was 20 mg Trolox/g, lower than our result.

A high correlation coefficient was observed between TPC and TFC with FRAP and CUPRAC methods. The correlation coefficients in these cases were 0.9752, 0.9825, and 0.9920, 0.9815 respectively. Moreover, the analysis revealed a significant positive correlation of CUPRAC with FRAP (0.9716).

Zhou and Yu [82] determined the correlation coefficient for CUPRAC, FRAP, and total phenolics in cauliflower genotypes. The results reported presented a significant correlation coefficient of CUPRAC with FRAP (0.711), CUPRAC with total phenolics (0.470), and FRAP with total phenolics (0.504). Deng et al. [83] also observed that total phenolic content and the measured antioxidant properties were correlated with each other. Sun and Tanumihardjo [84] also revealed a significant linear correlation among different antioxidant capacities (FRAP and TEAC) in a study with 56 vegetables. They have also observed a positive correlation among total antioxidant capacities and total phenolic content.

In this study, most of the analyzed byproducts showed high values of antioxidant activity by the FRAP and CUPRAC method; this is probably due to other structures that can function as ligands.

### 3.4. Development of the Relative Antioxidant Capacity Index RACI

The relative antioxidant capacity index (RACI) was developed as a statistical perspective by integrating food antioxidant capacity data determined by the several methods used. For obtaining the RACI values, it is necessary to determine the standard scores for each method. Standard scores obtained using different methods of analysis will have a similar contribution to the central trend of the average and can be compared without interference from different units, scales, and distributions [84]. The trend of the RACI value matched with the standard score from the four methods (Table 4).

**Figure 5:**
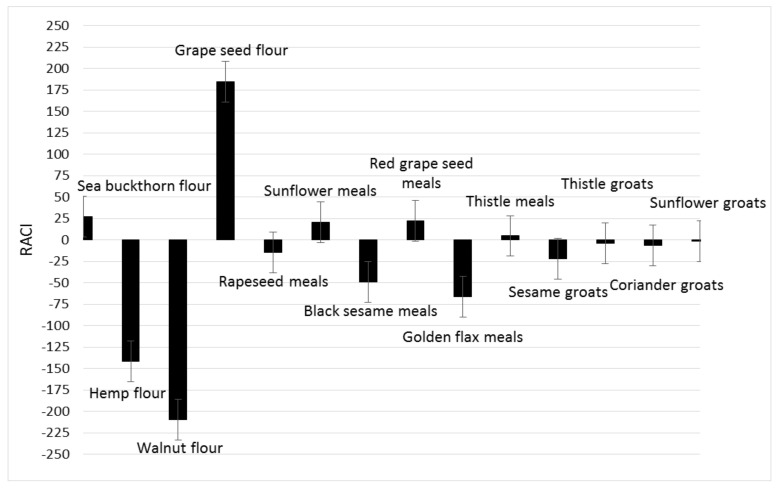
Relative antioxidant capacity index of 14 byproducts obtained in the vegetable oil industry.

In order to create a ranking of the antioxidant capacities of the analyzed byproducts, RACI was calculated (Figure 5).

**Figure 6:**
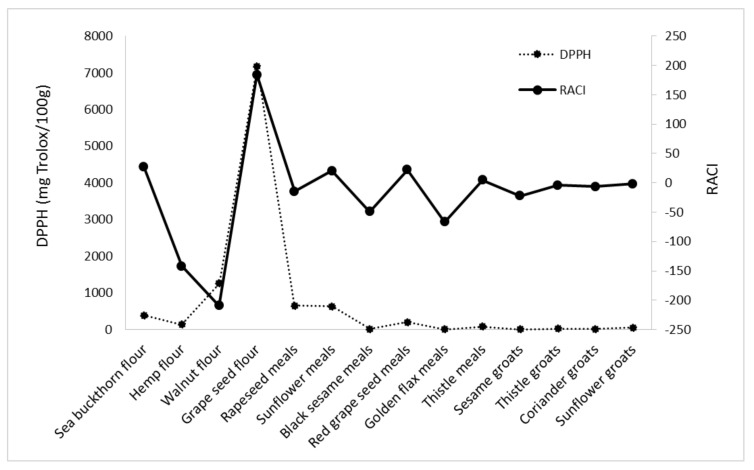
Correlation between RACI and DPPH activity.

**Figure 7:**
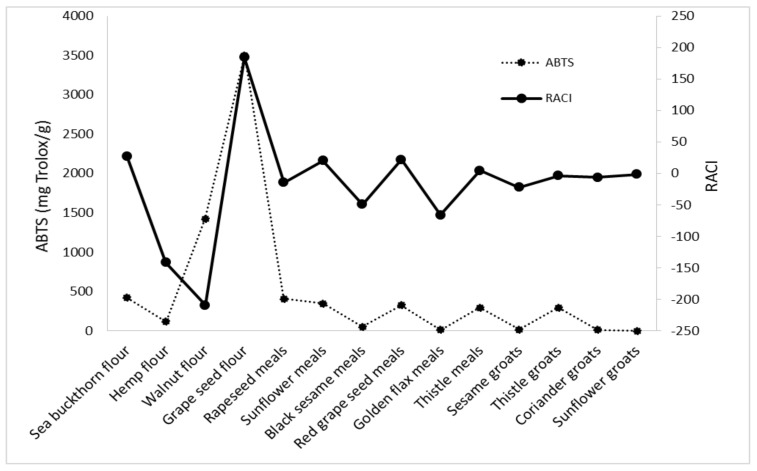
Correlation between RACI and ABTS activity.

**Figure 8:**
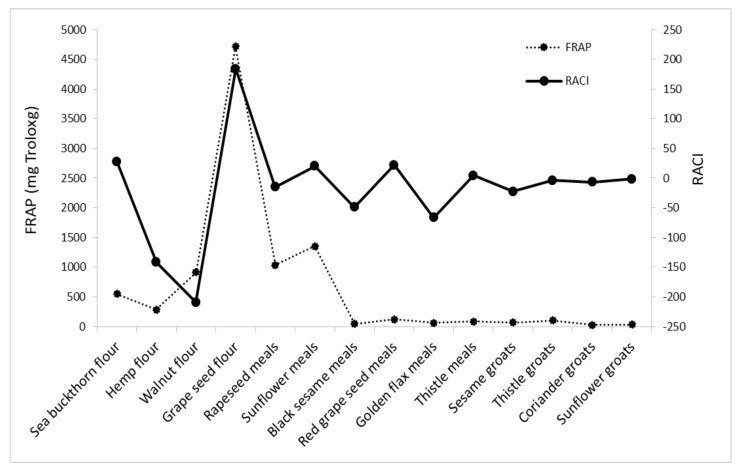
Correlation between RACI and FRAP activity.

**Figure 9:**
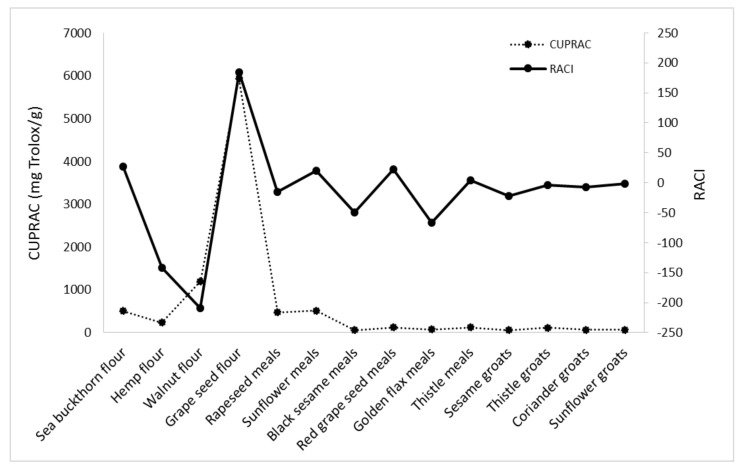
Correlation between RACI and CUPRAC activity.

RACI of analyzed byproducts is a scientific combination of data from different chemical methods with no unit limitation and no variance among methods. With this parameter, it is possible to make a much more precise comparison of the antioxidant capacities of the different food matrices. Based on the RACI value of the 14 byproducts obtained in the vegetable oil industry, the rank of antioxidant capacity ranged from −209.46 to 184.20. The highest relative antioxidant capacity index was observed in grape seed flour; the lowest was in walnut flour, with a value of −209.46. Each antioxidant activity used in this study was correlated with RACI. In this study, the most sensitive methods in developing RACI were FRAP and DPPH (r = 0.5795; r = 0.5766), followed by CUPRAC (r = 0.5578) and ABTS (r = 0.4449) (Figure 6, Figure 7, Figure 8 and Figure 9).

### 3.5. Determination of Antioxidant Capacity Using the PHOTOCHEM Device in an ACL System

In the present study, the ACL concentration was expressed as equivalent antioxidant activity in mg Trolox per gram of extract.

**Figure 10:**
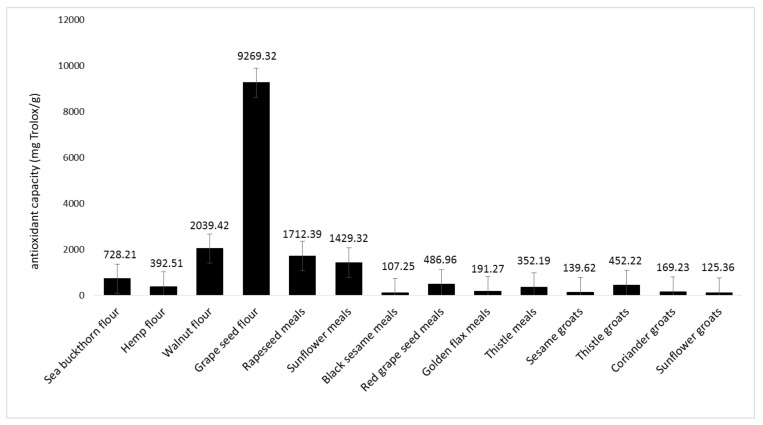
Antioxidant capacity using the PHOTOCHEM device in an ACL system.

The inhibition of photochemiluminescence (PCL) of luminol (5-amino-2,3-dihydro-l,4-phthalazinedione) in methanol by constituents of the phenolic-containing byproduct extracts was monitored using the PHOTOCHEM (Figure 10).

Lipid soluble antioxidant capacity data of the 14 byproducts obtained in the vegetable oil industry were between 107.25 and 9269.32 mg Trolox/g.

As can be seen in Figure 10, the highest value was determined in the grape seed flour sample. This value exceeded other measured data several-fold. The walnut flour (2039.42 mg Trolox/g), rapeseed meals (1712.39 mg Trolox/g), and sunflower meals (1429.32 mg Trolox/g) were the next samples with ACL values above 1000 mg Trolox/g. Obtained data for the black sesame meals, golden flax meals, sesame groats, coriander groats, and sunflower groats were the lowest at just over 100 mg Trolox/g. Senila et al. [85] analyzed total antioxidant capacity of four types of seeds (sunflower, flax, hemp, and sesame) using the photochemiluminescence assay. The antioxidant capacity varied between 7.5 and 112.5 mg Trolox/g. The results were lower than those presented in this study.

Sielicka et al. [86] analyzed six samples of flaxseed, walnut, rapeseed, pumpkin seed, evening primrose, and black cumin cold-pressed in terms of lipid-soluble antioxidant capacity analysis. They used hexane as the extraction solvent. The values ranged between 1.0 and 7.7 mM Trolox/L oil. The antioxidant capacity of flaxseed, walnut, and rapeseed pressed cold oils presented the lowest values.

Strong positive correlations between the antioxidant capacity of lipid-soluble compounds measured by photochemiluminescence assay (PCL) and the other methods used for determining antioxidant activity were found (Table 5).

It was observed that the highest correlation is between ACL and DPPH (r = 0.9952), followed by ACL and CUPRAC (0.9930). In order to determine the antioxidant capacity of lipid-soluble compounds of different cold-pressed oils, Sielicka et al. [86] used the photochemiluminescence and DPPH method. Similar to our results, they found a high correlation between these methods, with a correlation coefficient of 0.91.

All methods exhibited similar trends of antioxidant capacity of the analyzed byproducts obtained from the vegetable oil industry, but the obtained values differed, which might be attributed to the dissimilar measurement principles, as described by Prevc et al. [87]. In the PCL assay, optical excitation of a photosensitizer results in the generation of the superoxide radical, and then the ability to scavenge the radicals is evaluated by chemiluminescence. In contrast, the DPPH assay analyzes the ability to reduce the stable radical through determination of a decrease in absorbance. The CUPRAC assay measures the reducing power of antioxidants to convert cupric (Cu2+) to cuprous (Cu+) ion. The other cause of lower values of antioxidant capacity obtained by DPPH, CUPRAC, FRAP, and DPPH methods may be due to the use of ethanol instead of methanol.

It is known that the solvent used as an extraction medium can influence the degree of oxidation reaction [88]. Sielicka et al. [86] reported that pumpkin, sesame, rapeseed, and flaxseed oil samples dissolved in ethyl acetate presented much lower antioxidative capacity in the DPPH method than after solubilization of oils in other solvents.

## 4. Conclusions

In this study, the phenolic content, flavonoid content, and the lipid-soluble antioxidant capacity of 14 byproducts obtained in the vegetable oil industry were measured. Results confirm that the byproducts analyzed are a good source of many biological functional substances having considerable amounts of total phenolic content.

For determining the antioxidant activity, DPPH, ABTS, FRAP, and CUPRAC methods were used. The ACL method was used for determining the antioxidant capacity. The samples showed varied antioxidant capacities depending on the seed origin. In all methods performed, the highest antioxidant capacity was for the grape seed flour. Walnut flour, sunflower meals, and rapeseed meals are an excellent source of antioxidant substances with high antioxidant capacity. The polyphenol content and the antioxidant capacity of the byproducts from the vegetable oil industry are influenced by the variety and the method of obtaining the waste. The flour of the analyzed byproducts has a higher antioxidant activity than meals and groats. Photochemiluminescence analysis and DPPH, ABTS, FRAP, and CUPRAC assays were fully applicable to the evaluation of the antioxidant capacity of lipophilic fraction of byproducts obtained in vegetable oil industry samples, with correlation coefficients of 0.9952, 0.9735, 0.9874, and 0.9930, respectively. The results indicate that byproducts obtained from the vegetable oil industry (flour, meal, and groats) could be an inexhaustible source of phenolic compounds, especially flavonoids, with antioxidant properties as valuable functional ingredients with beneficial effects on human health. The byproducts obtained from the vegetable oil industry can be used as ingredients for new bakery products to improve their nutritional properties and antioxidant quality. Further studies are needed to determine the optimal concentration of the byproducts’ addition into wheat flour in order to achieve an improvement in the nutritional and sensory properties and to increase the antioxidant capacity of the bakery products.

## Figures and Tables

**Table 1:** Phenolic content and flavonoid content in the byproducts of the vegetable oil industry.

Sample Name	Total Phenolic Content	Total Flavonoid Content
	(Mg GAE/g)	(Mg QE/g)
Sea buckthorn flour	11.52 ± 0.50	9.28 ± 0.06
Hemp flour	4.68 ± 0.13	4.12 ± 0.07
Walnut flour	13.75 ± 0.24	7.57 ± 0.09
Grape seed flour	74.85 ± 0.43	51.24 ± 1.46
Rapeseed meals	11.24 ± 0.65	9.46 ± 0.50
Sunflower meals	11.70 ± 0.35	7.92 ± 0.14
Black sesame meals	3.88 ± 0.20	nd
Red grape seed meals	6.84 ± 0.67	nd
Golden flax meals	3.80 ± 0.28	nd
Thistle meals	7.97 ± 0.48	nd
Sesame groats	4.31 ± 0.32	1.41 ± 0.25
Thistle groats	8.39 ± 0.19	4.99 ± 0.37
Coriander groats	1.73 ± 0.09	nd
Sunflower groats	1.54 ± 0.03	nd

**Table 2:** Antioxidant activity of selected byproducts obtained in the vegetable oil industry.

Sample Name	DPPH	ABTS	FRAP	CUPRAC
Sea buckthorn flour	394.17 ± 1.50	419.46 ± 2.45	547.45 ± 13.14	503.43 ± 14.52
Hemp flour	139.59 ± 1.17	113.73 ± 13.47	285.81 ± 17.23	231.94 ± 18.74
Walnut flour	1257.49 ± 3.85	1423.98 ± 24.57	913.44 ± 15.19	1202.75 ± 23.99
Grape seed flour	7182.53 ± 6.12	3500.52 ± 66.45	4716.75 ± 131.88	5936.76 ± 96.42
Rapeseed meals	647.29 ± 1.36	406.55 ± 6.61	1034.92 ± 39.63	478.43 ± 30.88
Sunflower meals	628.58 ± 3.85	347.01 ± 20.97	1350.86 ± 72.20	510.49 ± 35.22
Black sesame meals	17.73 ± 1.04	48.81 ± 2.68	42.69 ± 2.75	63.31 ± 1.09
Red grape seed meals	200.77 ± 1.08	322.76 ± 27.81	119.92 ± 8.06	119.99 ± 5.54
Golden flax meals	9.25 ± 0.68	12.13 ± 0.61	61.54 ± 4.98	75.50 ± 8.02
Thistle meals	85.58 ± 3.29	292.81 ± 13.59	84.89 ± 6.18	125.75 ± 4.55
Sesame groats	7.58 ± 1.30	15.77 ± 5.05	66.21 ± 3.45	62.45 ± 2.38
Thistle groats	22.74 ± 1.74	293.14 ± 34.32	105.31 ± 12.41	112.54 ± 2.92
Coriander groats	17.64 ± 0.61	9.37 ± 2.22	26.47 ± 2.78	67.53 ± 0.95
Sunflower groats	55.06 ± 2.64	nd	34.46 ± 1.51	70.47 ± 2.96

All values are expressed as mg Trolox/g fresh weight.

**Table 3:** The correlation coefficients between total phenolic content and flavonoids with DPPH, ABTS, CUPRAC, and FRAP.

	DPPH	ABTS	FRAP	CUPRAC
TPC	0.9927	0.9660	0.9752	0.9920
TFC	0.9811	0.9477	0.9825	0.9815

**Table 4:** Standard scores of antioxidant capacity and RACI for the analyzed byproducts.

Sample Name	DPPH	ABTS	FRAP	CUPRAC	RACI
Sea buckthorn flour	43.15	36.74	16.59	11.98	27.12
Hemp flour	−475.64	−43.23	−23.81	−24.77	−141.86
Walnut flour	−521.89	−75.00	−154.93	−86.04	−209.46
Grape seed flour	685.39	7.71	13.11	30.58	184.20
Rapeseed meal	−20.75	−40.69	9.07	−6.38	−14.69
Sunflower meal	65.56	−1.39	13.50	3.81	20.37
Black sesame meal	−95.45	−25.44	−27.02	−49.25	−49.29
Red grape seed meal	79.20	7.46	0.58	0.86	22.03
Golden flax meal	−123.80	−133.28	−6.40	−2.24	−66.43
Thistle meal	−2.14	14.73	−1.25	7.28	4.65
Sesame groats	−60.11	−13.85	−5.65	−9.78	−22.35
Thistle groats	−33.97	6.16	1.89	10.51	−3.85
Coriander groats	−36.89	−13.86	−4.92	28.83	−6.71
Sunflower groats	0.66	0.00	−12.50	5.79	−1.51

**Table 5:** Correlation coefficients between ACL and DPPH, ABTS, FRAP, and CUPRAC.

Method	DPPH	ABTS	FRAP	CUPRAC
ACL	0.9952	0.9735	0.9874	0.9930

## Data Availability

The data presented in this study are available on request from the corresponding author. The data are not publicly available due to privacy or ethical restrictions.
